# South Korean validation of the COVID-related-PTSD scale in a non-clinical sample exposed to the COVID-19 pandemic

**DOI:** 10.1186/s40359-022-00844-2

**Published:** 2022-05-25

**Authors:** Hwa Jung Lee, Ye Jin Kim, Dong Hun Lee

**Affiliations:** Traumatic Stress Center, Department of Education, Sungkyunkwan University, Seoul, Republic of Korea

**Keywords:** COVID pandemic, Posttraumatic stress disorder checklist, PCL-5, COVID-related-PTSD, Validation, South Korea

## Abstract

The threat of COVID-19 outbreak in South Korea and around the globe challenged not only physical health but also mental health, increasing the chances of disorders such as posttraumatic stress disorder (PTSD). Such pandemic situation can be referred to a traumatic event for citizens. The present study aims to examine the psychometric properties of the PTSD Checklist (PCL-5), which is named the K-COVID-related-PTSD. The scale measures PTSD symptomology in the context of the COVID-19 pandemic in South Korea. A total of 1434 South Korean citizens were included in this study. The data were statistically analyzed using SPSS 21.0 and Mplus 8.0. The results of confirmatory factor analysis demonstrated a superior fit for the seven-factor hybrid model (*x*^2^ = 1425.445 (df = 149), CFI = 0.950, TLI = 0.937, SRMR = 0.033, RMSEA = 0.077) consisting of re-experiencing, negative affect, anxious arousal, dysphoric arousal, avoidance, anhedonia, and externalizing behaviors. Furthermore, the K-COVID-related-PTSD showed a satisfactory level of internal consistency (α = 0.793 to α = 0.939) with good convergent and discriminant validity. Finally, concurrent validity was confirmed by the significant correlations with all the negative mental health outcomes, such as PTSD symptoms, somatization, depression, anxiety, anger, negative affect, job burnout, and suicidal ideation. Overall, the current results demonstrate the K-COVID-related-PTSD is a valid scale and therefore has important implications for future pandemic-related studies.

## Introduction

The COVID-19 outbreak was declared a pandemic by the World Health Organization (WHO) on March 11, 2020, and the disease continues to cause significant damage worldwide. In response to the pandemic crisis, health organizations and ministries have adopted several non-pharmacological measures, such as social/physical distancing and lockdowns, isolation of COVID-19 positive and suspected COVID-19 patients, and quarantine of exposed individuals. While these efforts have reduced the spread of COVID-19, isolation and social distancing have negatively impacted the mental health of many individuals [1, 2]. Apart from the physical toll of the disease itself, individuals experience psychological distress due to traumatic stressors related to isolation, disturbed routines, and family and social life (e.g., loss of family and loved ones due to COVID-19) [3]. In particular, several studies have shown that individuals may experience the spread of COVID-19, and social distancing and self-quarantine measures are instituted to mitigate its spread as a traumatic stressor [4, 5]. Notably, trauma exposure is the primary etiologic risk factor for many mental illnesses, including posttraumatic stress disorder (PTSD).

PTSD refers to specific negative symptoms that might occur in individuals after exposure to one or more traumatic events [6]. Although the rate of PTSD in the general population is between 5 and 10%, its incidence can be as high as 45.9% among direct victims of disasters [7, 8]. During previous serious infectious disease outbreaks, the prevalence of PTSD ranged from 40 to 76%. A survey of survivors 3 years after the SARS epidemic in Hong Kong and China showed that over 40% of them displayed symptoms of PTSD [9]. In addition, results from a 1-year follow-up study of Ebola-infected patients in Sierra Leone documented a PTSD diagnosis rate of 76% [10]. With regard to South Korean samples, 41.7% of Middle East respiratory syndrome survivors displayed PTSD symptoms 12 months after their diagnosis [11]. As such, a pandemic of an unrecognized infection can be defined as a traumatic experience of acute and chronic effects at the individual and community levels. The fear of contagion and the risk of death for oneself and loved ones refers to a direct threat. In addition, indirect consequences were found to result in comorbid conditions including psychological distress, mood disorders, and general psychological symptoms of PTSD. Moreover, previous studies on the COVID-19 pandemic have found that a high risk of developing PTSD is not only valid in survivors, victim families, healthcare workers, and individuals with direct contact with infection, but also in the general population subjected to prolonged restrictive measures [12].

PTSD is classified as a type of trauma- and stressor-related disorder according to the Diagnostic and Statistical Manual of Mental Disorders, Fifth Edition (DSM-5). Specific criteria focused on identifying the causes and symptoms are required for the diagnosis of PTSD. PTSD can be diagnosed after exposure to a traumatic event and includes four specific dimensions (re-experiencing the trauma, avoiding reminders of the trauma, negative alterations in cognitions and mood, and alterations in arousal and reactivity) [6]. Following this criterion, the PCL-5 is one of the most studied screening instruments for adults at risk of developing PTSD. Initially, it was developed with four sub-factors (re-experiencing, avoidance, negative alterations in cognition and mood, and alterations in arousal). However recent PCL-5 studies have shown that PTSD symptoms can be described as having as many as six or seven factors [13–16]

The anhedonia model, as proposed by a Chinese study from a sample of the Wenchuan earthquake, has six factors: intrusion, avoidance, negative affect, anhedonia, dysphoric arousal, and anxious arousal [17]. This model separates negative alterations in cognition and mood factors into two distinct factors representing changes in negative versus positive affect. Similarly, the seven-factor hybrid model suggested by Armour et al. [18] was integrated from several six-factor models, distinguishing “externalizing behavior” as a factor in PTSD. This seven-factor hybrid model consisted of the following factors: re-experiencing, negative affect, anxious arousal, dysphoric arousal, avoidance, anhedonia, and externalizing behaviors. However, research on veterans showed mixed results, in which PTSD symptoms were derived as a single factor [19].

Based on these models, research on PCL-5 validation has been conducted in different countries in relation to various traumatic experiences and has compared different factor models based on their psychometric adequacy. Previous CFA studies have shown that the DSM-5 four-factor model is the best-fitting model in countries such as Brazil [20], Germany [21], Turkey [22], and Malaysia [23]. In addition, the six-factor model was found to fit well in Bangladesh (Islam et al. 2021) and the Netherlands [15], while the seven-factor model displayed a superior fit in France [13], Italy [12], and the Netherlands [15]. In South Korea, a previous validation study with Vietnam War veterans supported the superiority of the single-factor model [24], while the seven-factor hybrid model was found to be the most appropriate in another PCL-5 validation study with adults [25].

Several psychiatric comorbidities have been reported in disaster situations. Previous research has found that anxiety, depression, anger, and stress are common comorbid clinical conditions associated with PTSD symptoms. Moreover, suicidal thoughts and job burnout appear when continuously exposed to infectious and disaster situations [26–33]. However, we are unaware of any research that has examined the dimensional structure of the PCL-5 and its association with other mental health outcomes among South Koreans during the COVID-19 pandemic. Therefore, to confirm the validity of the Korean version of the PCL-5, the correlation between PTSD symptoms and related variables, as well as the K-COVID-related-PTSD scale, which measures PTSD symptoms, was analyzed. This study is important because it not only develops a Korean version of the COVID-related-PTSD scale but also estimates the total score that represents the overall severity of PTSD in Korean society due to the COVID-19 pandemic. Considering the unique characteristics of the COVID-19 pandemic in South Korea, this study aimed to examine the psychometric properties of the PCL-5.

The first goal of the current research was to assess the psychometric properties of the COVID-related-PTSD scale, which was designed to investigate the severity of PTSD symptoms in the South Korean population during the COVID-19 crisis. This assessment was performed by testing the scale’s factorial structure (one, four, six, or seven factors) using a CFA approach. The second goal was to examine its reliability and concurrent validity by exploring the relationship between COVID-related-PTSD and variables related to pandemic situations such as PTSD, somatization, stress, depression, anger, negative affect, fear, distrust, job burnout, satisfaction with life, and suicidal ideation.

## Method

### Participants

This study used a national sample by implementing an online survey based on the South Korean population census standard in 2018, including age, gender, and residential area. Initially, 2440 individuals entered the survey and 988 participants who did not meet the criterion of the present study or did not complete the survey were excluded, indicating completion rate of 59.5%. Lastly, 18 participants were excluded due to careless responses by screening partially random or inattentive data. Thus, a total of 1434 participants were used for the final analysis. Among the total sample, 731 (51%) were men and 703 (49%) were women. The mean age of the participants, who ranged from 19 to 84 years of age, was 44.34 (SD = 13.93). All participants met the following eligibility criteria: They were able to read and write Korean proficiently, were able to provide informed consent, and were aged 19 years or older. Table 1 provides detailed sociodemographic information as well as COVID-19-related information of the total sample.

**Table 1:** Sociodemographic and COVID-19-related information of the participants (N = 1434)

Variables	*N* (%)
Sex	
Men	731 (51.0)
Women	703 (49.0)
Age	
19–29 years	275 (19.2)
30–39 years	267 (18.6)
40–49 years	317 (22.1)
50–59 years	319 (22.2)
60–69 years	219 (15.3)
> 70 years	37 (2.6)
Occupation	
Student	121 (8.4)
Office worker	731 (51.0)
Medical practitioner	53 (3.6)
Self-employed	157 (10.9)
Housewife	205 (14.3)
Unemployed	121 (8.4)
Others	47 (3.3)
Residential area	
Capital area	695 (48.4)
Other metropolitan area	289 (20.0)
Medium and small sized cities	450 (31.6)
Socio-economic level	
Upper middle class	56 (3.9)
Middle class	602 (42.0)
Lower middle class	776 (54.1)
Household type	
One-person household	228 (15.9)
Group household	1206 (84.1)
COVID-19-related experiences	
Similar symptoms	48 (3.3)
Cohort isolation	2 (0.1)
Quarantine	35 (2.4)
Infected	2 (0.1)
No symptoms	1347 (93.9)
COVID-19-related experiences of family and acquaintances	
Symptoms similar to COVID-19	75 (5.2)
Cohort isolation	7 (0.5)
Quarantine	157 (10.9)
Infected	68 (4.7)
No symptoms	1127 (78.6)

### Procedure

The survey was conducted via an Internet survey company between February 19 and March 3, 2021. The number of confirmed COVID-19 cases and deaths in South Korea during the survey period were 91,236 and 1612, respectively. During this period, government regulations that mandated social distancing, banned private gatherings of more than five individuals, and ensured that restaurants and bars were closed after 10 pm were in place in the nation’s capital area. In addition, the AstraZeneca and Pfizer vaccination campaigns had begun in Hong Kong, Nepal, the United States, and Japan for high-risk groups (e.g., people with chronic diseases, medical staff, and older adults). The participants were assured that their data would remain confidential and anonymous, and their informed consent for participating in this study was subsequently taken. The survey took approximately 20–30 min to complete, and online credit points of around 3 US dollars that can be converted into cash were provided to the participants as compensation. The questionnaire consisted of two sections. The first section asked about participants’ sociodemographic information, including sex, occupation, residential area, socio-economic level based on the OECD standards of the middle class, and household types as well as COVID-19-related experiences. The second section included the PCL-5 and different sociological and psychological scales. The survey company is certified by ISO 9001, indicating that it meets the most recognized quality management system standards. To ensure the security of the survey, the company used a firewall (WAF) and the DigiCert security service. Moreover, all survey responses were collected through an encrypted secure socket layer (SSL), which enabled authentication, encryption, and decryption of data. Finally, all data were removed securely once the operation of the system expired. The current study was approved by the Institutional Review Board (IRB) of the university to which the researchers were affiliated, and all methods were performed in accordance with the relevant guidelines and regulations.

### Measures

#### PCL-5

To measure the level of the participants’ PTSD symptoms, we used the Korean version [34] of the PTSD Checklist (PCL-5) [35] which later applied the diagnostic criteria of the DSM-5 [36]. The PCL-5 has 20 items, and the sub-factors are re-experiencing (five items; e.g., “painful and unwanted memories about the stressful experience repeatedly come to mind”); avoidance (two items; e.g., “avoiding memories, thoughts, or emotions related to the stressful experience”); negative alterations in cognition and mood (seven items; e.g., “difficulty remembering important parts of the stressful experience”); and hyperarousal (six items; e.g., “nervousness, anger, externalizing behavior, or explosive/aggressive behavior”). Responses are provided using a 5-point Likert scale that ranges from “not at all” (0 points) to “very much” (4 points). Higher scores indicate more severe PTSD symptoms. According to a recent South Korean PCL-5 validation study by Lee et al. [25], Cronbach’s alpha coefficients were 0.90, 0.87, 0.91, and 0.92 for re-experiencing, avoidance, negative alterations in cognitions and mood, and hyperarousal, respectively. In this study, the PCL-5 showed good internal consistency with Cronbach’s alpha coefficients of 0.93, 0.88, 0.90, and 0.91, and composite reliability (CR) of 0.94, 0.88, 0.90, and 0.91 for re-experiencing, avoidance, negative alterations in cognitions and mood, and hyperarousal, respectively.

#### K-PC-PTSD

In this study, we used the Korean version of the PC-PTSD-5 (K-PTSD-5) scale developed by Yum [37] to screen for PTSD symptoms. Originally, the PC-PTSD-5 scale was developed by Prins et al. [38] and revised by Prins et al. [39]. The K-PTSD-5 consists of five items as a single factor, with items scored dichotomously as either “yes” (1 point) or “no” (0 point). Higher scores indicate a higher risk of symptoms, and the cutoff point for high-level PTSD symptoms was estimated to be 3. The Cronbach’s alpha coefficient of K-PC-PTSD-5 was 0.73, and in this study was 0.66.

#### Somatization

The revised Patient Health Questionnaire (PHQ-15) by Kroenke, Spitzer, and Williams (2002) was used to assess the pattern and severity of the physical symptoms [40]. The PHQ-15 consists of 15 items extracted from the PHQ [41]. Each item is scored on a 3-point Likert scale ranging from “not bothered at all” (0 points) to “very distressed” (2 points). The cumulative score ranged from 0 to 45, with a higher score indicating a higher level of physical symptoms. Cronbach’s alpha coefficient of the Korean version of the PHQ-15 was 0.73 [42]. In this study, Cronbach’s alpha coefficient was 0.87.

#### Depression

The Center for Epidemiological Studies Depression Scale (CES-D), a self-reporting simple screening test tool developed by the American Institute of Mental Health (1971) and validated by Radloff [43], was used to examine the participants’ level of depression. The scale was originally validated by Cho and Kim [44] and the short Korean version of the CES-D-10 was standardized by Shin [45]. The scale consists of ten items, and participants were asked to answer questions pertaining to the symptoms of depression experienced over the past week, with either “yes” (1 point) or “no” (0 points). The cutoff point that indicated a significant level of depression was estimated to be 3. According to Shin [45], Cronbach’s alpha coefficient was 0.79. In this study, Cronbach’s alpha coefficient was 0.83.

#### Anxiety

The Generalized Anxiety Scale (GAD-7), developed by Spitzer et al. [46] and later validated in Korean by Seo and Park [47], was used to identify the anxiety level of the participants and probable cases of generalized anxiety disorder. Seven items that asked about participants’ anxiety and worries related to the COVID-19 crisis were rated using a 4-point Likert scale ranging from “not at all” (0 points) to “nearly every day” (3 points). A higher total score indicates a higher severity of anxiety symptoms, with an optimal cutoff point of 5. Of a total score of 21, 5 or more, 10 or more, and 15 or more are classified as mild, moderate, and severe anxiety symptoms, respectively. In the validation study conducted by Seo and Park [47], the Cronbach’s alpha coefficient was 0.92. In this study, it was 0.93.

#### Posttraumatic anger

The Dimensions of Anger Reactions-5 (DAR-5) scale, which was developed by Forbes et al. [48], was used to measure the level of anger symptoms. It was first translated into Korean by bilingual researchers and later back-translated by a professor of counselling and Ph.D. researchers. Discrepancies were noted and discussed until the final version was completed. It has five items: frequency, intensity, duration, aggression, and interference with social relations. On the original scale, participants were asked to respond while recalling their daily lives over the past 4 weeks. However, in this study, participants responded while thinking about the difficulties they experienced in their daily lives during the COVID-19 pandemic to measure individual anger symptoms. The participants responded using a 5-point Likert scale ranging from “none of the time” (1 point) to “all of the time” (5 points). Higher scores reflected worse anger symptoms. Cronbach’s alpha coefficient for all items of the original DAR-5 was 0.90, indicating a high level of reliability. In this study, Cronbach’s alpha coefficient was 0.91.

#### Negative affect

The Positive and Negative Affect Schedule (PANAS) scale, which was developed and validated by Hong [49] based on circumstances in Korea, was used. The PANAS is a widely used checklist that reflects two subscales containing 11 items of positive affect and 11 items of negative affect. Each item is scored on a 5-point Likert scale ranging from “not at all” (1 point) to “very much” (5 points). As this study aimed to measure the negative affect of citizens during the COVID-19 crisis, 11 items of negative affect were extracted for use. Cronbach’s alpha coefficient of the Korean version of the PANAS [49] was 0.90. In this study, Cronbach’s alpha coefficient was 0.93.

#### Job burnout

We used the Maslach Burnout Inventory-General Survey (MBI-GS) developed by Schaufeli [50] to measure job burnout. The original MBI-GS consists of 16 items, including five items that measure exhaustion, five items that measure cynicism, and six items that measure professional efficacy. A validation study of the South Korean version [51] that consisted of only 15 items was conducted, and the remaining item was translated and back-translated by Ph.D.-level researchers. All items are scored on a seven-point scale; higher scores on exhaustion and cynicism and lower scores on professional efficacy indicate a higher level of burnout. In Shin’s [51] study, Cronbach’s alpha coefficients for exhaustion, cynicism, and professional efficacy were 0.90, 0.81, and 0.86, respectively. In this study, Cronbach’s alpha coefficients for exhaustion, cynicism, and professional efficacy were 0.92, 0.90, and 0.92, respectively.

#### Suicidal ideation

To assess the degree of suicidal ideation, a Korean validation study [52] of the Depressive Symptom Inventory-Suicidality Subscale (DSI-SS), a subscale of the Hopelessness Depression Symptom Questionnaire [53], was used. The items were about the frequency, intensity, controllability, and content of the suicidal thoughts. Each item is rated on a 4-point Likert scale (0–3 points), and the total score ranges from 0 to 12 points. Higher scores are indicative of greater severity of suicidal ideation. Cronbach’s alpha coefficient of the Korean version of the DSI-SS was 0.93. In this study, Cronbach’s alpha coefficient was 0.95.

### Data analysis

Descriptive statistics were used to analyze the participants’ characteristics, and a normality test was subsequently conducted to determine if the data followed a normal distribution, followed by item-total correlation analysis. Subsequently, CFA was conducted to evaluate four potential structural models of the K-COVID-PTSD scale based on theoretical and empirical evidence of PTSD. First, a single-factor model, in which all items were loaded onto one general factor, was tested. The DSM-5 four-factor model, which included re-experiencing, avoidance, negative alterations in cognition and mood, and hyperarousal, was tested next. We then examined the third model, a six-factor anhedonia model that consisted of re-experiencing, negative affect, anxious arousal, dysphoric arousal, avoidance, and anhedonia. Finally, we tested a seven-factor model suggested by Armour et al. [18], which included re-experiencing, avoidance, negative affect, anhedonia, externalizing behavior, anxious arousal, and dysphoric arousal. A CFA was conducted using maximum likelihood (ML) estimation. Model fit indices were examined using the chi-square test, comparative fit index (CFI), Tucker-Lewis index (TLI), standardized root mean square residual (SRMR), and root mean square error of approximation (RMSEA). 0.90 or higher for the TLI and CFI values is regarded as a satisfactory fit [54], and lower SRMR values are desirable [55]. Values of < 0.08 are within the acceptable range for RMSEA, and values of ≤ 0.06 show a close fit to the data. Additionally, the Akaike information criterion (AIC) and Bayesian information criteria (BIC) indices were used to compare the different models [56, 57]. Next, the reliability analysis was followed by a Cronbach’s alpha analysis. Next, the reliability analysis was followed by a Cronbach’s alpha analysis. A Cronbach’s alpha value of 0.7 and above is considered to indicate good internal reliability. Additionally, convergent validity was assessed by composite reliability (CR) and average variance extracted (AVE). The acceptable value of CR and AVE is 0.7 and 0.5, respectively [58, 59]. The discriminant validity through chi-square difference analysis was assessed. The differences larger than 3.84 indicate a significant level of discriminant validity. Lastly, the concurrent validity of the scale was verified via Pearson correlations between measures of PTSD, somatization, depression, anxiety, posttraumatic anger, negative affect, job burnout, and suicidal ideation, respectively. The data were statistically analyzed using SPSS 21.0 and Mplus 8.0.

## Results

The normality test was performed by calculating the mean, standard deviation, skewness, and kurtosis. The skewness ranged from 314 to 1.371, and the kurtosis ranged from − 0.956 to 0.738. The values of both skewness and kurtosis were below the absolute values of skewness ( ≤|2.0|) and kurtosis ( ≤|4.0|) [60], indicating that the items followed a normal distribution. Also, all items must be properly correlated with the total scale to maintain the scale's homogeneity while utilizing the measurement tool. As indicated in Table 2, correlations varied from 0.621 to 0.810 (*p* < 0.01), which deemed appropriate, indicating a value over 0.50.

**Table 2:** Correlation between total scale and each item

Item	Correlation
1	.784**
2	.768**
3	.804**
4	.810**
5	.807**
6	.756**
7	.735**
8	.757**
9	.790**
10	.771**
11	.774**
12	.621**
13	.649**
14	.750**
15	.777**
16	.744**
17	.738**
18	.783**
19	.729**
20	.670**

**p < .01

In the CFA, the parameters for the measurement model were estimated using the ML method with Mplus 8.0. The fit of each model was evaluated using SRMR, RMSEA, and TLI, which are indices that favor simplicity without being affected by the sample size, and CFI, a goodness-of-fit index that is less sensitive to the sample size and measures the error of the model (see Table 3). An RMSEA and SRMR of less than 0.08, and a CFI and TLI of 0.90 or more are considered to be adequate model fits. The CFA revealed that the value of the one-factor model was 4866.417 (*df* = 170, *p* < 0.001; CFI = 0.818, TLI = 0.796, RMSEA = 0.139, and SRMR = 0.066), indicating that the model was inadequate. The CFA of the DSM four-factor model presented a value of 2678.033 (*df* = 164, *p* < 0.001); CFI = 0.902, TLI = 0.887, RMSEA = 0.103), indicating that this model was also inadequate. The six-factor model showed adequate CFI, TLI, and SRMR values of 0.902, 0.925, and 0.035, respectively, but inadequate RMSEA value of 0.084. However, the value of the seven-factor model was 1425.445 (*df* = 149, *p* < 0.001; CFI = 0.950, TLI = 0.937, RMSEA = 0.077), indicating adequate to good fit indices. Additionally, the AIC value (63,648.541) for the seven-factor model was lower than that of the one-factor (67,047.512), four-factor (64,871.128), and six-factor models (63,948.969), indicating a better comparative fit. In addition, considering the BIC value, the model with the lowest absolute value of BIC became the optimal model [61], indicating that the seven-factor model showed the lowest BIC index, with the difference being greater than 10 [57]. On the basis of these considerations, a seven-factor model was selected. Confirmation of the factor loadings revealed that the factor loading of the items was 0.5 or more in all models, which was also appropriate (see Table 3).

**Table 3:** Confirmatory factor analysis factor models of the Korean version of the COVID-related-PTSD (K-COVID-related-PTSD)

K-COVID-related-PTSD items	Single-factor model		DSM-5 four-factor model		Six-factor anhedonia model		Seven-factor model	
	Factor	Factor loading	Factor	Factor Loading	Factor	Factor loading	Factor	Factor Loading
1. Disturbing memories of the experience	1	0.823	1	0.867	1	0.868	1	0.867
2. Disturbing dreams of the experience	1	0.809	1	0.860	1	0.863	1	0.863
3. Suddenly feeling as if the stressful experience was actually happening again	1	0.845	1	0.900	1	0.901	1	0.902
4. Upset when reminded of stressful experience	1	0.842	1	0.869	1	0.867	1	0.866
5. Physical reactions to reminders of the experience	1	0.841	1	0.863	1	0.863	1	0.863
6. Avoiding memories, thoughts, or feelings related to experience	1	0.788	2	0.905	2	0.903	2	0.904
7. Avoiding external reminders of the stressful experience	1	0.765	2	0.869	2	0.871	2	0.870
8. Trouble remembering the experience	1	0.785	3	0.773	3	0.799	3	0.799
9. Negative beliefs of self, other people, and the world	1	0.809	3	0.820	3	0.839	3	0.839
10. Blaming self or others for the experience	1	0.794	3	0.802	3	0.821	3	0.821
11. Having strong negative feelings such as fear, horror, anger, guilt, or shame	1	0.774	3	0.795	3	0.772	3	0.772
12. Loss of interest in activities	1	0.601	3	0.649	4	0.764	4	0.768
13. Feeling distant or cut-off from other people	1	0.627	3	0.679	4	0.819	4	0.822
14. Trouble experiencing positive feelings	1	0.732	3	0.775	4	0.870	4	0.866
15. Irritability, angry outbursts, or acting aggressively	1	0.774	4	0.841	6	0.843	5	0.890
16. Taking too many risks or doing things that could cause you harm	1	0.756	4	0.804	6	0.797	5	0.861
17. Being superalert, watchful, or on guard	1	0.735	4	0.809	5	0.837	6	0.834
18. Feeling jumpy or easily started	1	0.781	4	0.854	5	0.888	6	0.892
19. Having difficulty concentrating	1	0.714	4	0.792	6	0.796	7	0.860
20. Trouble falling or staying asleep	1	0.661	4	0.705	6	0.711	7	0.766
*χ*^2^ (*df*)	4866.417 (170)		2678.033 (164)		1737.874 (155)		1425.445 (149)	
CFI	0.818		0.902		0.939		0.950	
TLI	0.796		0.887		0.925		0.937	
SRMR	0.066		0.049		0.035		0.033	
RMSEA	0.139		0.103		0.084		0.077	
AIC	67,047.512		64,871.128		63,948.969		63,648.541	
BIC	67,363.605		65,218.831		64,344.086		64,075.267	

*CFI* comparative fit index, *TLI* Tucker–Lewis fit index, *SRMR* standardized root mean residual, *RMSEA* root mean square error of approximation, *AIC* akaike information criteria

The items of each model of K-COVID-related-PTSD exhibited high internal reliability (see Table 4). The Cronbach’s alphas for the subscales were all good and adequate, considering the single-factor model (Cronbach’s α = 0.965), the DSM-5 four-factor model (Cronbach’s α = 0.881–0.939), and the seven-factor model (Cronbach’s α = 0.793–0.939). In addition, the correlations between the subscales of the selected seven-factor model fell within the recommended level, ranging from 0.524 to 0.792 [62]. Overall, this reflected the independence and multidimensionality of each subscale. Additionally, the AVE and CR were checked to verify the convergent validity of the seven-factor model, which was found to be the most suitable for the factor structure of K-COVID-related-PTSD. The results showed that the CR values were all higher than the AVE values, with values higher than 0.7, indicating good convergent validity of the scale [58, 63].

**Table 4:** Internal reliabilities, convergent validity, and correlations between subscales of K-COVID-related-PTSD (N = 1434)

	Re-experiencing	Avoidance	Negative affect	Anhedonia	Externalizing behaviors	Anxious arousal	Dysphoric arousal
AVE	.706	.787	.653	.672	.767	.767	.663
CR	.935	.881	.881	.881	.868	.854	.797
α	.939	.881	.880	.863	.865	.853	.793
Full scale	.910**	.819**	.931**	.800**	.836**	.843**	.804**
Re-experiencing	–	.792**	.846**	.592**	.705**	.687**	.620**
Avoidance		–	.770**	.524**	.586**	.616**	.557**
Negative affect			–	.685**	.739**	.723**	.687**
Anhedonia				–	.656**	.662**	.696**
Externalizing behavior					–	.764**	.659**
Anxious arousal						–	.726**
Dysphoric arousal							–

**p < .01

As a result of conducting a chi-square difference test to confirm discriminant validity, the difference between all sub-factors of K-COVID-related-PTSD showed a value much higher than the 3.84 threshold, indicating each subscale acting as a distinct concept (see Table 5). Overall, these reflect the independence and multidimensionality of each subscale.

**Table 5:** Discriminant validity via chi-square difference test (N = 1434)

	1	2	3	4	5	6	7
1. Re-experiencing	–						
2. Avoidance	338.185	–					
3. Negative affect	252.506	267.539	–				
4. Anhedonia	1137.851	903.037	570.846	–			
5. Externalizing behavior	511.547	693.729	332.869	473.956	–		
6. Anxious arousal	495.541	537.68	321.763	391.117	136.897	–	
7. Dysphoric arousal	375.941	411.737	223.644	154.873	238.279	93.699	–

The significant correlations were found between K-COVID-related-PTSD and other related variables, as well as the CI value of each correlation. Regarding the effect size, the value fell between 0.103 and 0.408, interpreted as small to moderate level. As presented in Table 6, The K-COVID-related-PTSD and its subscales displayed a strong positive correlation with PTSD symptoms, somatization, depression, anxiety, and anger. Additionally, the full scale and its subscales displayed a comparatively low positive correlation with negative affect, job burnout, and suicidal ideation.

**Table 6:** Correlations coefficients between the full scale/subscales and other variables (N = 1434)

Variable	Total score (CI)	Intrusion (CI)	Avoidance (CI)	Negative mood (CI)	Anhedonia (CI)	Externalizing behavior (CI)	Anxious arousal (CI)	Dysphoric arousal (CI)
PTSD	0.542 (.504–.578)	0.504 (.464–.542)	0.453 (.411–.493)	0.485 (.444–.524)	0.457 (.415–.497)	0.388 (.343–.431)	0.483 (.442–.522)	0.432 (.389–.473)
Somatization	0.556 (.519–.591)	0.478 (.437–.517)	0.424 (.381–.466)	0.494 (.454–.532)	0.474( (.433–.513)	0.424 (.381–.466)	0.508 (.469–.545)	0.541 (.503–.577)
Depression	0.630 (.598–.660)	0.555 (.518–.590)	0.453 (.411–.493)	0.554 (.517–.589)	0.536 (.498–.572)	0.548 (.511–.583)	0.552 (.515–.587)	0.585 (.550–.618)
Anxiety	0.639 (.607–.669)	0.566 (.530–.600)	0.502 (.462–.540)	0.573 (.537–.607)	0.514 (.475–.551)	0.537 (.499–.573)	0.572 (.536–.606)	0.567 (.531–.601)
Posttraumatic anger	0.635 (.603–.665)	0.537 (.499–.573)	0.452 (.410–.492)	0.580 (.545–.613)	0.529 (.491–.565)	0.625 (.592–.656)	0.569 (.533–.603)	0.541 (.503–.577)
Negative affect	0.490 (.450–.528)	0.398 (.354–.441)	0.392 (.347–.435)	0.436 (.393–.477)	0.471 (.430–.510)	0.374 (.329–.418)	0.432 (.389–.473)	0.451 (.409–.491)
Job burnout	0.420 (.376–.462)	0.333 (.286–.378)	0.321 (.274–.367)	0.359 (.313–.403)	0.423 (.380–.465)	0.328 (.281–.373)	0.361 (.315–.405)	0.417 (.373–.459)
Suicidal ideation	0.490 (.450–.528)	0.464 (.422–.504)	0.365 (.319–.409)	0.451 (.409–.491)	0.391 (.346–.434)	0.453 (.411–.493)	0.406 (.362–.448)	0.372 (.327–.416)

*CI* confidence interval

## Discussion

The outbreak of the COVID-19 pandemic worldwide was a traumatic event that challenged individuals’ physical and mental health, highlighting the importance of managing disorders such as PTSD. The aim of this study was to examine the psychometric properties of the PCL-5 scale, which investigates PTSD symptomology, in the context of the COVID-19 pandemic in South Korea. The results revealed that the seven-factor COVID-related-PTSD scale exhibited superior fit indices with good internal reliability, good convergent and discriminant validity, and concurrent validity, thereby demonstrating that it is psychometrically sound and culturally relevant.

The results of this study supported the seven-factor model by comparing it with the single-factor, four-factor, and six-factor models in the context of COVID-19. Equivalent factor models were extracted in Italy and China during the COVID-19 outbreak [12, 64]. However, based on studies on the use of the PCL-5 in relation to various kinds of traumatic events such as transportation accidents, exposure to war, financial crises, and bereaved experiences, varying factor solutions were yielded. Other studies with individuals who were injured in car and motor vehicle accidents [23, 65] and individuals who were exposed to lifetime traumatic events [20–22] found the four-factor model to be the best fit. A South Korean study on PCL-5 with Korean veterans of the Vietnam War supported a one-factor model [24]. However, it is difficult to generalize the results because of the specificity of the study sample. Recently, a study conducted with the South Korean national survey data supported a seven-factor model, which is in line with the results of the present study even though the type of traumatic event differed [25]. Moreover, many recent PCL-5 studies have shown that PTSD symptoms can be further subdivided into six- or seven-factor models [13–16]. Considering the previous results of both the original PCL-5 scale and pandemic-specific scale, the results of the present study appear to be reasonable. Furthermore, K-COVID-related-PTSD demonstrated good convergent and discriminant validity. As suggested by Ashbaugh et al. [13], the seven-factor model best describes and covers all the PTSD symptoms with each subscale independently measuring according to the targeted content.

The concurrent validity of the K-COVID-related-PTSD was satisfactory in that all of the variables (PTSD, somatization, depression, anxiety, traumatic anger, negative affect, job burnout, and suicidal ideation) were positively correlated with the complete scale as well as all seven subscales. This result is consistent with the previous validation studies of the PCL-5, which illustrated that negative psychological variables were closely related to and coexisted with PTSD symptoms [12, 22, 66, 67]. Moreover, previous studies have found that variables such as job burnout and suicidal ideation are closely related to pandemic-induced PTSD symptoms [68, 69], which is consistent with the present study.

The present study has several limitations that should be considered. First, it relied on a single self-report measure. To overcome this limitation, additional assessment methods, such as structured interviews or observational measures, are recommended to ensure the validity of the data. Second, despite the large sample size, the cross-sectional nature of the study limited the inference of causal relationships between variables. Therefore, longitudinal research that considers different pandemic-related contexts, such as the vaccination rate, social distancing rate, and severity of the pandemic, would serve to further validate the scale. Third, as the study utilized a nonclinical sample, future studies with clinical samples diagnosed with psychopathologies should be replicated to improve the validity of the study. If possible, cross-cultural studies would lead to a broader understanding of this scale.

Despite these limitations, the findings in this study appear to be critical in establishing effective therapeutic approaches during and after similar disastrous situations. Such unexpected pandemic outbreaks can cause devastating psychological outcomes at the individual and community level. Therefore, further use of the tool to understand the predictors in the context of pandemics is recommended.

## Conclusions

To our knowledge, the present study is the first to translate and evaluate the psychometric properties of COVID-related-PTSD in South Korea, considering the COVID-19 pandemic as a traumatic event. The results showed that the K-COVID-related-PTSD is a valid and reliable instrument for screening PTSD symptoms during the COVID-19 pandemic. The COVID-19 pandemic has been estimated to not be an isolated event but a calamitous event that bears a high possibility of reoccurring even after resolution. Therefore, measuring the consequences of a pandemic could be useful in preparing for similar future situations. Another key strength of the present study is that it used a nationwide sample, which can be interpreted as being more representative of the South Korean population. Future studies should not only confirm the results of the present study but also examine other facets of mental health and trauma within the context of the pandemic.

## Data Availability

The datasets used and/or analyzed during the current study are not publicly available due to the datasets currently being used for ongoing research, but are available from the corresponding author upon reasonable request.

## Abbreviations

AIC: Akaike information criterion
AVE: Average variance extracted
BIC: Bayesian information criteria
CES-D: Center for Epidemiological Studies Depression Scale
CFA: Confirmatory factor analysis
CFI: Comparative fit index
CI: Confidence interval
CR: Composite reliability
DAR: Dimensions of Anger Reactions
DSI-SS: Depressive symptom inventory-suicidality subscale
DSM-5: Diagnostic and Statistical Manual of Mental Disorders, Fifth Edition
EFA: Exploratory factor analysis
GAD: Generalized Anxiety Scale
IRB: Institutional Review Board
MBI-GS: Maslach Burnout Inventory-General Survey
ML: Maximum likelihood
PANAS: Positive and Negative Affect Schedule
PHQ: Patient Health Questionnaire
RMSEA: Root mean square error of approximation
SRMR: Standardized root mean square residual
SSL: Secure socket layer
TLI: Tucker Lewis index
